# Stochasticity and homeostasis in the *E. coli* replication and division cycle

**DOI:** 10.1038/srep18261

**Published:** 2015-12-16

**Authors:** Aileen Adiciptaningrum, Matteo Osella, M. Charl Moolman, Marco Cosentino Lagomarsino, Sander J. Tans

**Affiliations:** 1 FOM Institute AMOLF, 1098 XG Amsterdam, the Netherlands; 2 Dipartimento di Fisica and INFN, University of Torino, V. Pietro Giuria 1, Torino, I-10125, Italy; 3 Department of Biochemistry, University of Oxford, Oxford OX1 3QU, United Kingdom; 4 Sorbonne Universités, UPMC Univ Paris 06, UMR 7238, Computational and Quantitative Biology, 15 rue de l'École de Médecine, Paris, France; 5 CNRS, UMR 7238 Paris, France

## Abstract

How cells correct for stochasticity to coordinate the chromosome replication and cellular division cycle is poorly understood. We used time-lapse microscopy and fluorescently labelled SeqA to determine the timing of birth, initiation, termination, and division, as well as cell size throughout the cell cycle. We found that the time between birth and initiation (B-period) compensates for stochastic variability in birth size and growth rate. The time between termination and division (D-period) also compensates for size and growth variability, invalidating the notion that replication initiation is the principal trigger for cell division. In contrast, the time between initiation and termination (C-period) did not display such compensations. Interestingly, the C-period did show small but systematic decreases for cells that spontaneously grew faster, which suggests a coupling between metabolic fluctuations and replication. An auto-regressive theoretical framework was employed to compare different possible models of sub-period control.

For the cell cycle to progress properly, chromosome replication, metabolism, and cellular division must be coordinated^1^. At the same time, various cell-cycle events display significant intrinsic cell-to-cell variability. In *E. coli* for instance, the interdivision time, the time between birth and replication initiation, and the size at birth have been shown to vary by up to 30% for isogenic cells growing under constant conditions^2^,^3^,^4^,^5^. Stochasticity in the replication and division cycle could lead to premature division before chromosome replication is terminated, or excessively large cell sizes if division is delayed until replication is completed. Variability could amplify over time^6^, as exponential growth implies that cells that are born large grow faster because they contain more active components, hence yielding daughters that are even larger. These findings raise the question whether and how cells compensate for stochastic variability to coordinate the replication and division cycle and to maintain homeostasis^7^,^8^,^9^.

Classic studies of *E. coli* physiology have shown inherent links between replication and cell division. Using observations on *Salmonella* cell size and growth rate by Schaechter, Maaloe, and Kjeldgaard^10^ and on the DNA replication pattern in *E.coli* by Cooper and Helmstetter^11^, Donachie proposed that DNA replication is initiated at a critical mass per replication origin - independently of the available nutrients and resulting growth rate^12^. Changes in the timing of this early step of the cell cycle would logically equally affect all subsequent cell-cycle events including division, and hence allow for size control over a wide range of growth rates. While direct measurement of the initiation mass is challenging and has not been without debate, this model agrees with diverse experimental results^13^ and successfully predicted a regulator serving as a proxy for cell-size^14^, though other control mechanisms have also been proposed^15^,^16^,^17^. Importantly however, these findings concern changes in mean cell-cycle parameters in response to changes in external conditions or mutations, rather than cellular responses to stochastic variability in constant environments.

Whether cells adjust cell-cycle parameters in response to stochastic variability, in order to coordinate the replication and division cycle is incompletely understood. It has been shown that *E. coli* interdivision time can compensate for stochastic variability in birth-size^18^, with recent analyses indicating that division is triggered by the accumulation of a fixed cellular mass or volume^19^,^20^. How division variability relates to replication variability, and how this affects size homeostasis, remains an open question. Compensation for cell size variability could be realized by a stochastic variant of the initiation-mass model: cells with birth-sizes above the population average would then initiate early, divide early, and hence produce smaller daughters. However, cells that are born large due to stochasticity are physiologically different from cells that are born large because of external nutrients. Hence, it remains unclear whether the initiation-mass model is relevant to stochastic variability in birth size^19^,^20^. Another cause of stochastic variability is metabolic activity and growth, which was recently found to fluctuate significantly^21^,^22^. *A priori*, control of initiation mass would only seem partially effective in compensating for such growth fluctuations, as growth varies at timescales smaller than the cell-cycle^21^ and thus also after initiation. Elucidating this possible dynamic interplay between replication, division, and growth, is essential to understanding cell cycle control and the maintenance of homeostasis.

Here, we present a single-cell method to monitor the replication and growth cycles simultaneously in single slow-growing cells, using time-lapse microscopy. We use fluorescent labeling of the SeqA^23^ protein to visually monitor the formation and disappearance of the chromosomal replication forks in real-time. SeqA proteins bind to newly synthesized hemimethylated DNA and dissociate when it becomes fully methylated^24^, and thus label the DNA in the wake of the replication fork. Phase-contrast imaging in combination with image analysis algorithms provides measurement of the cell size throughout the cell cycle. This approach allows one to estimate the timing of replication and division events, the cell-size throughout the cell cycle, as well as the rate of growth for individual cells. In turn, this allows one to quantify the variability of these parameters and their cross-correlations that inform on compensatory adjustments.

## Results

### Replication and division in single cells

**Figure 1:**
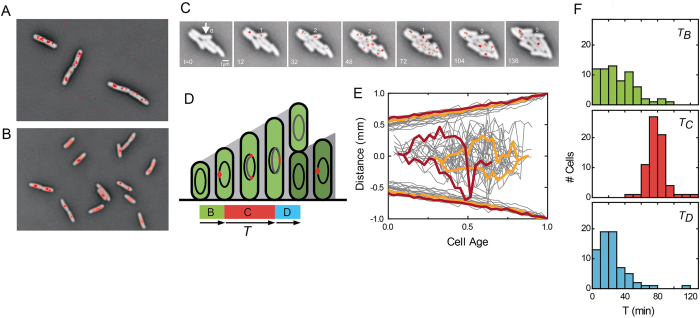
Replication and division variability in single cells. (**A**) Cells grown rapidly in defined rich medium. SeqA is fluorescently labeled with mCherry (red). Foci report on replication forks. (**B**) Cells grown slowly in succinate minimal medium. (**C**) Time-lapse images of cells growing on succinate. (**D**) Schematic representation of the chromosomal replication cycle in slow-growing cells. Red indicates the hemimethilated DNA binding SeqA. Indicated are the time between birth and replication initiation (B period), the subsequent time until termination (**C** period), and the subsequent time until division (D period). (**E**) Position of SeqA-foci and cell length against cell age, with 0 denoting birth and 1 division. Two typical cell cycles are highlighted in color. Top and bottom lines indicate cell length determined from phase contrast images. Middle lines indicate position of SeqA foci along the cell axis. N = 81 cell cycles. F) Corresponding histograms of B,C, and D periods.

To follow chromosome replication dynamics in single cells we expressed mCherry-labelled SeqA proteins from a plasmid at non-induced (leakage) expression levels, in addition to the endogenous SeqA. The cells were grown in exponential phase in defined rich medium with doubling time of 29.5 minutes. The latter was similar to the doubling time of cells only expressing endogenous SeqA (29.9 minutes). Fluorescence microscopy indicated multiple SeqA foci in the former (Fig. 1A). The observed average of 3.5 foci per cell is an underestimate, as not all foci could be distinctly identified. In a succinate MOPS minimal medium yielding a doubling time of about 130 minutes, the cells typically displayed either 1 or 2 foci, or no distinct foci but rather a diffuse fluorescence signal throughout the cell (Fig. 1B). On average the cells had 1.3 foci per cell. In the following we focused on the slow medium because the foci could be better identified. The findings are qualitatively consistent with previous studies of SeqA in individual cells^25^,^26^, and the occurrence of multiple nested replication forks in cells with division times under 1 hour^11^, though other studies did not observe cells without SeqA foci at slow growth^27^,^28^. These differences between observed numbers of SeqA foci are unclear, but could be attributable to differences in growth conditions and genetic background.

The slow-growing cells were followed by time-lapse microscopy, as they grew exponentially into microcolonies of about 30 cells (Fig. S1). We note that the interdivision time and size distribution of the cells was similar as for WT cells with only endogenous SeqA (Fig. S2). Phase contrast and fluorescence images were taken every 4 minutes and analyzed with custom algorithms, which yielded estimates for the time of birth (*T*_*birth*_) and division (*T*_*div*_) for each cell cycle, and SeqA foci were tracked manually. At birth, no foci were typically observed until one SeqA focus appeared, which subsequently elongated and separated into 2 nearby foci (Fig. 1C–E). The distance between foci first increased and then decreased again until a single focus was observed, which subsequently disappeared nearing cell division. This pattern is consistent with current replication models, where replication is initiated by two replication forks that co-localize at the replication origin, which then replicate bidirectionally, and end up at the terminus leading to replication termination^29^. The fluorescence imaging was in some images limited by imperfect focusing, overlap between foci, or background fluorescence from unbound SeqA. We focused our analysis on the cells that displayed a clear pattern in the number of foci throughout their complete cell cycle (54% of the 150 cell cycles from four experiments). We did observe one other clear pattern in some cells (0-1-2-1-2-0), though only rarely (<1%), which could indicate a separation of the termini while still hemimethylated. After replication, termini have been shown^30^,^31^ to remain co-localized for significantly longer than the time required to fully methylate the DNA (of order minutes^32^), consistent with our observation of a single disappearing SeqA focus.

Next, we combined the division and replication information. The time between birth and first SeqA focus was taken as an estimate of the B-period, the subsequent time until the disappearance of the last SeqA focus was taken as the C-period, and the subsequent time until division as the D-period (see Fig. 1D). On average, we found values of 30, 78, and 25 min. for the B, C, and D periods respectively, and hence a division time *T*_*d*_ of 133 min. Where previous estimates of the B, C, and D periods were for instance limited by the fitting of distributions obtained by FACS analysis^3^, in our method the hemimethylation and hence SeqA foci may persist beyond replication termination, potentially leading to an overestimation or different demarcation of the C-period. However, chromosomal DNA remains hemimethylated for minutes only^32^, which is smaller than the time-resolution of the fluorescence images, and SeqA binding affinity drops 100-fold upon full methylation^24^. Some error also occurs in determining the moment of division, though we estimate it to be not more than 10 minutes. Nonetheless, our mean values for the B, C, and D periods are in approximate agreement with previous work (Fig. S3), which for this growth rate can be interpolated as 28 min., 67 min., and 38 min. respectively^3^. Note that systematic errors that affect the mean are less relevant to the variabilities and correlations that we investigate here.

The measured B, C, and D periods displayed significant variability between cells (Fig. 1F). The B period was broadly distributed (CV = 0.7, Fig. 1F), which indicates a large variability in the timing of initiation, in line with previous studies that employed pulse-labelling with radioactive thymidine in synchronized cultures^2^. In some cells, replication initiated just after division, which could indicate that the initial trigger occurred in the mother cell. The C period was comparatively narrowly distributed (CV = 0.16, Fig. 1F). This lack of variability of *T*_*C*_ is consistent with the processive nature of the chromosome replication process, which can result in reduced timing variability^3^,^33^. The distribution of the D period was broad again (CV = 0.6). Some D periods were negligible, which could mean that the moment of replication termination was overestimated due to a persistence of SeqA foci. An alternative explanation is that constriction had started before replication termination, which would mean that the two are not as tightly coupled as previously suggested^9^.

### Cell size compensation

**Figure 2:**
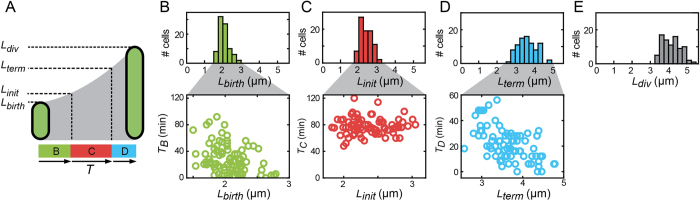
Compensation for size variability. (**A**) Schematic diagram indicating the cell cycle periods, and the corresponding length of the cell at birth (L_birth_), replication initiation (L_init_), termination (L_term_), and division (L_div_). (**B**) Histogram of cell lengths at birth, and correlations with the B period. N = 81 cell cycles. (**C**) Histogram of cell lengths at initiation, and correlations with the C period. D) Histogram of cell lengths at termination, and correlations with the D period. E) Histogram of cell lengths at division.

The size of the cells at birth displayed a significant variability, with lengths ranging from 1.5 to 2.8 μm and a CV of 0.14, consistent with previous work^34^. To explore whether the timing of the various cell-cycle events compensate for this variability, we computed cross-correlations between cell size and duration of the B, C, and D periods (Fig. 2). We found a significant correlation between the size at birth and the B period (*c*_*p*_ = −0.36, *p* < 0.05, Fig. 2B). Thus, cells that were born small on average had a larger B-period. Note that in the absence of such *L*_*birth*_-*T*_*B*_ correlations, the CV of the cell-length at the end of the B period would have increased because of the additional variability in *T*_*B*_. Here, the negative *L*_*birth*_-*T*_*B*_ correlations suppress this increase and even produce a slight decrease (CV = 0.12). These data suggested that the timing of initiation is modulated in response to variations in birth size within a population. We also found that *T*_*B*_ *was* strongly correlated with *T*_*d*_ (*c*_*p*_ = 0.79, *p* < 0.05). This suggests that the variations in replication timing have an effect down stream on the division cycle as a whole. For instance, the large B period of a small-born cell on average results in a comparatively large overall *T*_*d*_, consistent with the idea that the modulation of replication initiation timing helps to control cell division. As such, the data suggested a stochastic variant of Donachie’s mass model.

In contrast to the B-period, the C-period displayed no correlation with cell-length at the start of that period (Fig. 2C). The cell-size variability should thus increase during the C-period, though not by much given the comparatively small variability of *T*_*C*_. Indeed, at the end of the C-period the cell-length variability had increased somewhat (CV = 0.14), which confirms that the C-period does not contribute to suppressing variability in cell-size.

The D-period displayed an anti-correlation with the cell-length at the start of the period (*c*_*p*_ = 0.45, *p* < 0.05, see Fig. 2D). Like for the B-period, this negative correlation implied a cell-size correction, as comparatively small cells at the end of the C-period had a longer D-period on average. This finding suggested that the timing of division is affected by mechanisms that are independent from B-period corrections. The negative *L*_*term*_-*T*_*D*_ dependence suggested that cell-size variability could be suppressed during the D-period, though significant variability in *T*_*D*_ for a certain *L*_*term*_ (Fig. 2D) could also increase cell-size variability. Overall, cell-length variability did not increase but rather decreased slightly during the D period (CV = 0.12 at division). We note that *T*_*D*_ was not correlated significantly with birth size (p-value for correlation is 0.97), suggesting that the additional size-correction mechanism for the D-period duration acts on variability of the accumulated size during the current cell cycle and is not due to memory of previous cell cycles.

### Growth rate compensation

**Figure 3:**
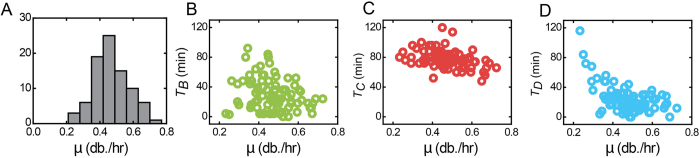
Compensation for growth rate variability. (**A**) Histogram of growth rates for individual cell cycles. The values are determined by fitting the logarithm of the cell length against time, and thus indicate a length doubling per hour. N = 81 cell cycles. (**B**) B period against growth rate. (**C**) C period against growth rate. (**D**) D period against growth rate.

Not only stochasticity of replication and division events, but also variability in the rate of growth can potentially contribute to cell-size variability. We determined the elongation rates (*μ*) of individual cells as the slope of the logarithm of the cell length over time from birth to division. The mean elongation rate over all cells 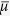 was found to be 0.47 db/hr, which is consistent with the previously determined *T*_*div*_ of 133 min. The variability in 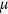 values was quantified by a CV of 0.22 (Fig. 3A). We found that the B, C, and D periods all displayed negative correlations with growth rate (*c*_*p*_ = −0.25; −0.39; −0.55 respectively, *p* < 0.05, Fig. 3B–D), consistent with trends observed for population-mean values obtained in bulk with different growth media (Fig. S3). Slow-growing outliers contributed to the correlation with *T*_*D*_, but after removing the 6 slowest growing cells the correlations remained significant (*p* < 0.05). *T*_*D*_ appeared to level off to a constant value for the faster growing cells, as also seen in bulk (Fig. S3C).

A negative correlation of the B-period with growth is in line with a stochastic critical mass model, as the critical mass is reached earlier for higher growth rates. Variations in *T*_*B*_ then not only compensate for variability in birth size, but also for variability in growth rate. The correlation between growth rate and birth size was not significant (*p* = 0.88), which suggests that they represent two independent sources of variability. The negative correlation between the D-period and growth (Fig. 3D) indicated a similar compensation: cells with higher growth rates reached the critical division length earlier on average. Overall, a stochastic version of the critical mass model can qualitatively explain the correlations with growth rate: cells that happen to grow fast or are born large achieve the critical mass earlier. See below for a quantitative comparison.

The magnitude of the C-period modulation with growth rate is small compared to the B and D periods (Fig. 3B–D). Over the observed growth rate range (0.2 to 0.7 db./hr) the observed *T*_*C*_ decreases significantly however (from 90 min to 60 min). Thus, the rate of chromosome replication appears affected by the rate of growth. This finding indicates that not only replication initiation, but also the process of replication itself is coupled to cellular growth. This coupling may have regulatory causes. However, it could also have a metabolic origin, with replication being limited by components such as nucleic acids, which can be more abundantly available at higher growth rates.

### Cell cycle homeostasis model

**Figure 4:**
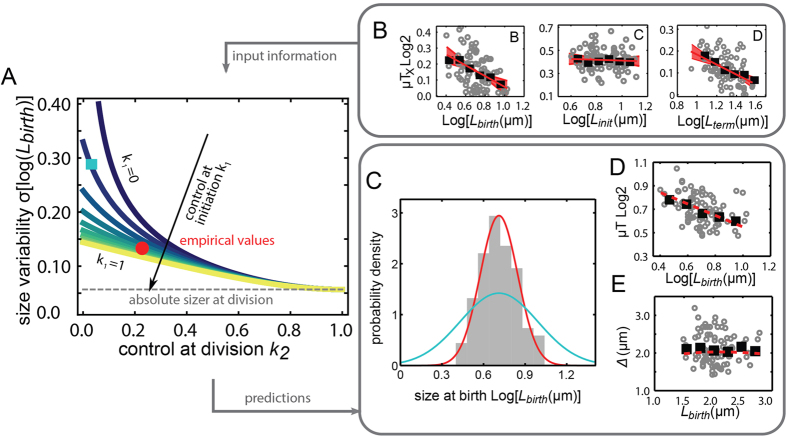
Model and comparison with data. (**A**) Width of the cell size distribution, and its dependence on the strength of control in the B period (*k*_*1*_) and the D period (*k*_*2*_). In contrast, the C period is not modulated in response to size variability, and hence acts as a pure timer (panel B). Red circle indicates the empirically determined values. Decreasing *k*_*1*_ and *k*_*2*_ leads to a widening distribution (cyan square, panel C). See SI Appendix for model details. (**B**) Values of *k*_*1*_ (left) and *k*_*2*_ (right) are estimated from the empirical data. *μ* indicates the exponential elongation rate, *T*_*X*_ the duration of the sub-period *X. X* can be either B, C, or D. The C period data is consistent with a timer (center). Black squares are averages of binned data; red line is the best linear fit; shaded region represents the standard error of the fit parameters (confidence level of 95%). N = 81 cell cycles. (**C**) Comparison between data and predicted birth size distribution. Using the fits of panel B, the predictions (red line) are consistent with the empirical histogram of birth sizes (histogram). For lower compensation strengths, the distribution is wider (cyan line, square in panel A). (**D**) Model predictions (dashed red line) for the dependence of total cell elongation on birth size is consistent with the data. E) Model predictions for the added size as a function of birth size. The three sub-periods together yield an approximately constant average added size (dashed red line), which is consistent with the empirical data.

The above analysis underscores the need for stochastic models of the cell cycle that describe the division cycle that consider the coupling between cell-cycle events. We employed a theoretical framework describing cell cycle as an auto-regressive discrete-time stochastic process^35^,^36^ that includes sub-periods (Fig. 4, SI appendix). The aim was to provide insight into how overall cell-size variability depends on the noise and strength of compensation within each cell-cycle period, and to assess the requirements for convergence to a steady-state size distribution. Extending a previously introduced formalism^36^, each sub-period is either characterized by controlling the duration, or the size at the end of the period, or anything in between (SI appendix). Specific cases include a stochastic variant of Donachie’s constant initiation-mass model^12^, in which the B period displays size control, and a ‘sub-period adder’ model in which a constant size is added within the sub-periods.

First, the analysis indicated that a steady-state size distribution cannot be obtained by controlling the durations of the sub-periods. Even if control is very precise, and all period durations are highly reproducible from generation to generation, the remaining small timing errors accumulate over time resulting in divergent size-distributions. One may alternatively consider that the fold-change in size during the period is controlled to a particular value. These scenarios closely resemble duration-control, and hence also do not yield steady-state size distributions (SI appendix). Finally, cells may adjust period duration, and hence the net growth in that period, in response to size variability.

In our model, the strength of size compensation for the B- and D-periods are denoted as *k*_*1*_ and *k*_*2*_ respectively, while the C period does not display size compensation (SI appendix). We find that *k*_*1*_ and *k*_*2*_ are 0.34 and 0.23 respectively (Fig. 4B), which are in between a size control that fully compensates for size variability (*k* = 1) and a time control that does not compensate for size variability (*k* = 0, see also SI Appendix). As expected, the compensation strength in the C period is negligible (Fig. 4B). Using these values (Fig. 4A, red point), we find the model properly predicts the width of the birth size distribution (Fig. 4C, red line). The overall relation between birth size and size-change over the full cycle is also well predicted (Fig. 4D,E). This correspondence is not obvious a priori, as hidden correlations between parameters could have produced deviations. Hence, the model indicates which elements are sufficient to describe how the variability of the division cycle can be understood in terms of the variability and control within the underlying sub-periods.

The model clarifies a number of other issues. First, for low but non-zero values for both *k*_*1*_ and *k*_*2*_, we find that the size distribution is wide but non-divergent (Fig. 4A). Thus, a weak size-compensation in just one of the sub-periods is sufficient to achieve size homeostasis with a wide size distribution. Second, when *k*_*2*_ is high, increasing *k*_*1*_ does not significantly reduce the distribution width, while for intermediate and low *k*_*2*_, increases in *k*_*1*_ does help to narrow the size distribution (Fig. 4A). In contrast, when *k*_*1*_ is high, increasing *k*_*2*_ does further narrow the size distribution (Fig. 4A). This asymmetry between *k*_*1*_ and *k*_*2*_ originates from the temporal order of the cell cycle, and the accumulation or errors: even if initiation compensates perfectly, additional variability can accumulate downstream, while for perfect division control at the end of the cycle, the earlier initiation modulation matters less. Third, we use it to quantitatively compare our data and model parameters with two alternatives: i) A ‘sub-period adder’ model in which a constant size is added during the sub-periods, following recent reports of a constant added size between birth and division^19^,^20^. ii) A stochastic variant of Donachie’s constant initiation mass model. Here, the B-period is characterized by size-control, and the C- and D-periods by time control. Within our framework, this scenario corresponds to *k*_*1*_ = 1 and *k*_*2*_ = 0.

In the sub-period adder model (i), the average size added in each sub-period is constant and hence does not depend on the size at the start of the period (Fig. S3A–C). However, the data does show a correlation in each sub-period (*p* < 0.05). In the B period it decreases, consistent with the negative *T*_*B*_ - *L*_*birth*_ correlation (Fig. 2B). In the C period it increases slightly, consistent with a constant *T*_*C*_ (Fig. 2C) and exponential growth. In the D period it decreases, consistent with the negative *T*_*D*_ - *L*_*term*_ correlation (Fig. 2D). The stochastic initiation-mass model (ii) is consistent with the observed trend of decreasing added size for larger born cells (Fig. S3A). However, the constant initiation size that it assumes does not agree with the data (Fig. S3D), showing that consistency in the B period is only qualitative. This model assumes that the C period is a ‘timer’, i.e., that its duration is independent on other parameters such as the size at the beginning of the period, which does describe the data (Fig. 2C, S3B). It also assumes a fixed duration for the D period, which is inconsistent with the data (Fig. 2D, S3C). Interestingly however, note that this model would yield a birth length variability that is identical to the observed one (S3F, blue square and red point).

Thus, none of these two alternative models properly describes the sub-period data. Rather, in the B periods one observes a partial size compensation, which can be seen as being in between initiation-size and adder models (Fig. 4, Fig. S3). The C period is characterized by time control, and control of the D period resembles that of the B period, as also reflected in our model. The size added during the full cycle is compatible with an adder model (Fig. 4E). Thus, having sub-periods with incomplete size-control and sub-periods with time control can together give rise to an added size that is constant on average. However, the added size over the full cell cycle varies significantly (between 1.5 and 3 micron), and hence is not strictly controlled. Note that thus far, adder and constant initiation-size models have been used for different situations than considered here: initiation-size models for population-mean cell sizes (which vary for different external conditions), and adder models for the added size during the full cell cycle.

## Discussion

Here we studied variability in replication and division events in single *E. coli* cells under constant conditions, as well as the correlations between them. To do so, we monitored replication using SeqA labeling. Previous still images of SeqA foci, using both immunostaining and GFP-labeling, suggested that each SeqA focus could represent either one^26^,^37^ or two^38^,^39^,^40^,^41^ replication forks. The time-lapse imaging indicated that one SeqA focus forms initially, then splits into two separating foci, which finally merge again and disappear. This spatio-temporal pattern is consistent with a single initiation per cycle and two spatially separated replication forks. Growth and division was followed with concurrent phase-contrast imaging. In this manner we followed key parameters in the growth and replication cycle, and quantified correlations between them. A set of autoregressive models in which the different cell-cycle sub-periods can both induce noise and correct for it, was used to determine the elementary ingredients for homeostasis and to interpret the data.

Previous studies have studied the *E. coli* cell cycle at the population level, and hence do not address coordination between replication and division within single cells. Moreover, whereas the former concern cell-cycle changes in response to nutrient conditions and genetic background, and hence involve important adjustments in cellular composition and metabolism, the latter result from stochasticity of cell cycle events and within the growth machinery at fixed nutrient conditions^21^. Bulk studies have shown that replication initiation is controlled by the ratio between the active form of DnaA (DnaA-ATP) and the inactive form (DnaA-ADP), which are interconverted in a replication dependent manner^42^. However, the dynamics of active and inactive forms of DnaA throughout the cell cycle has not been investigated experimentally at the single-cell level.

The single-cell data we obtained indicates that as the cell cycle progresses, stochasticity in growth rate and timing of initiation and division events continuously generates variability. Importantly, the time between birth and replication initiation (B period) is modulated to compensate for stochastic variability. As such, these findings suggest a stochastic variant of the constant initiation mass model^33^. By delaying initiation, cells that happen to be born comparatively small or grow slowly can initiate at a size closer to the population mean. However, this compensation is partial and hence the mean size at initiation is not a constant, but rather remains positively correlated with birth size variability. We furthermore note that these findings are not inconsistent with the idea of cell division licensing the origins for initiation (suggested by previous single-cell measurements)^30^, as our method does not allow for the analysis of growth regimes that support two subsequent initiations within the same cell cycle.

The time between termination and division (D period) also displayed compensatory effects. Within initiation-mass models, the D period is generally assumed not to compensate for size variability, and hence would not depend on size. Here we found that the D period does display such compensation for stochastic size variability. These results support the idea that initiation is not the only or principal trigger of division control^29^,^43^. A possible candidate for such a control mechanism has been hypothesized^44^, and involves an accumulating factor necessary to initiate cell division. This factor may be the constriction ring protein FtsZ^9^, with SlmA inhibiting FtsZ polymerization in concert with nucleoid segregation^45^,^46^. Spatio-temporal oscillations of the MinCDE system may also favor specific ‘wavelengths’, and hence distinct division lengths^47^.

Compared to the B and D periods, the time between initiation and termination (C period) was both less variable and was not modulated by cell size. This is consistent with the view of DNA replication as a processive process that is not actively modulated once started. Thus, the C period can be characterized by time control. Growth rate variations did affect the C period, with faster cells completing the C period in a shorter amount of time. While the precise origin is unclear, this finding is consistent with bulk observations (Fig. S4), and suggests that stochastic variations in growth and replication are dependent on a common factor. For instance, a transient limitation in metabolites and associated metabolic reallocation could impact both the rate of replication and of growth, or vice versa, replication arrests could impact growth. Such a coupling would be in line with the observation that fluctuations in common compounds can affect growth and protein expression^19^. Nutrient-dependent regulation of *E. coli* cell size has been reported previously^43^. During growth under nutrient-rich conditions, the OpgH protein localizes to the septal site, delaying division by interacting with the Z-ring. The data do not seem to be consistent with a scenario in which growth depends strictly on the number of ribosomes, as they should not affect the rate of DNA replication.

To gain mechanistic insight into the role of sub-periods in achieving cell-size homeostasis, we employed a stochastic modeling framework using autoregressive processes. This analysis indicated that size-homeostasis cannot be achieved by precise control of time or fold-changes in size. The latter is relevant to mechanisms that exploit dilution by volume growth. This can be understood intuitively: small multiplicative errors here accumulate as in a random walk, and hence produce ever-broadening size distributions. We find that such a lack of size control is observed for the C period that acts as a timer. We find that small size compensations in just a single sub-period are sufficient to achieve size homeostasis, though the width of the distribution can then be exceedingly wide. Increased compensation strengths are important to narrowing the size distribution, which is for instance relevant to limiting wasteful growth. We find that the B- and D periods both display intermediate compensation strengths, rather than perfect compensation in the B period as suggested by the constant initiation mass model. Thus, while the total added size between divisions does not depend on birth size, consistent with recent reports^19^,^20^, the same does not hold for the sub-periods. Rather, The B and D periods can be thought of as compensating for the lack of control in the C period, resulting in an added size between divisions that is variable but on average independent on birth size.

Our findings show that within the *E. coli* cell cycle, parameters of the replication and division cycle are adjusted not only in response to the external environment, but also to stochastic internal processes, in order to maintain homeostasis. The cell cycle is expected to couple to various other aspects of cellular growth and physiology, which may also have unknown stochastic components. The approach followed here may be used to explore the dynamic coordination of these phenomena.

## Additional Information

**How to cite this article**: Adiciptaningrum, A. *et al.* Stochasticity and homeostasis in the *E. coli* replication and division cycle. *Sci. Rep.* **5**, 18261; doi: 10.1038/srep18261 (2015).

## Supplementary Material

Supplementary Information
