# Supplementary material for: Stochasticity and homeostasis in the E. coli replication and division cycle
Source: Sci Rep. 2015 Dec 16;5:18261. doi: 10.1038/srep18261 (PMC4680914; doi:10.1038/srep18261)
Supplement: Supplementary Information [file srep18261-s1.pdf]

Stochasticity and homeostasis in the E. coli replication and division cycle

Supporting Information for Adiciptaningrum *et al.*

Aileen Adiciptaningrum, Matteo Osella, Charl Moolman,
Marco Cosentino Lagomarsino, and Sander J. Tans

CONTENTS

S1. Methods 1
A. Strains and growth conditions 1
B. Microscopy sample preparation 2
C. Microscopy 3

S2. Minimal models of division control 4
A. Background 4
B. Timer arguments for cell-size homeostasis. 5
Simple considerations on size homeostasis for cell-cycle sub-periods 7

S3. Generic model for three cell cycle sub-periods 9
A. General setting 9
B. Compatibility between model and data 12

References 13

S1. METHODS

A. Strains and growth conditions

Experiments were performed on strain ASC215 [1], which is the *E. coli* K-12 strain ASC129 (*fimA::gfpmut2*) transformed with plasmid pASC215. Plasmid pASC215 was constructed by fusing *seqA* gene from pGAP40 [2] to the N-terminus of mCherry [3] fluorescent protein, using a linker composed of five amino acids (Glu-Phe-Asn-Asn-Asn), which is under the control of a repressed trc down promoter, and insertion into vector pSAV047 (Amp^R, pBR322 ori; *rop*$^-$). Cells

were grown at 37°C on EZ Defined Rich Medium (Teknova) and MOPS medium (Teknova) supplemented with 0.4% sodium succinate (Sigma), supplemented with 100μ g/mL ampicilin (Sigma Aldrich). For microcolony growth, MOPS agar slab was made by mixing hot 2% agaroseMP (Roche) solution to concentrated medium as mentioned above. All liquid cultures were grown aerobically. Before imaging, cells were grown overnight. The next day, the overnight culture was diluted with pre-warmed medium and grown for another 5 hours to be in the exponential phase (to an OD of about 0.02). When a rich medium was used, the dilution was done twice, each 1000X and grown for 2.5 hours. When a minimum medium was used, the dilution was done once and such that the OD of the culture were not exceeding 0.02 in 5 hours of growth.

B. Microscopy sample preparation

Two microscope slides, one with a hole of size 18 mm x 55 mm, were attached to each other by use of silicon grease (Dow Corning) and heated to 80°C. Next, 10X concentrated medium was mixed with hot agaroseMP solution to its final concentration (total volume is 1 mL) and spread horizontally across the center of the microscope slides cavity (Figure S1). Immediately afterwards, the microscope slides were transferred to a cool flat surface, and covered with a 24 mm x 60 mm silanized coverslip. Silanized coverslip was made using RepelSilane (Amersham Biosciences). The coverslip was dropped right on top of the liquid agar so that the agar surface would become flat upon cooling. The agar solidified at room temperature in 2-3 minutes.

When the colour of the agar had become less transparent, the slides were incubated in the microscopes 37°C incubation chamber to avoid any cold shock to the cell when they were deposited on the agar surface. Inside the incubation chamber, the silanized coverslip was carefully removed, revealing a thin, flat agar slab. Excess agar slab was trimmed to leave a thin strip of agar of dimension about 2 mm x 55 mm across the centre of the cavity. The rest of the cavity served as air reservoir for cell growth. By use of pre-warmed pipette tip, 2-3 tiny droplets of exponential liquid culture were dropped on top of the agar strip. The sample was then closed immediately by dropping a pre-warmed normal coverslip on top of the cavity and the gap between the coverslip and the microscope slide was sealed with silicon grease. Over time, each single cell grew into a

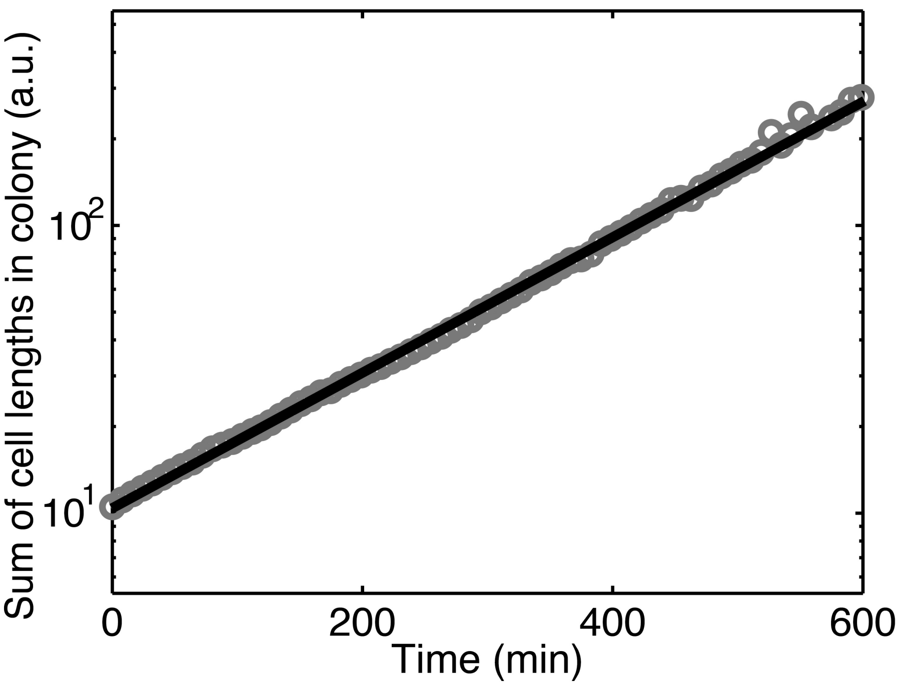

Supplementary Figure S1. **Exponential growth of microcolony.** The sum of the lengths of all cells within a microcolony is plotted against time. The data indicates the microcolony grows exponentially at a rate of 0.48 db/hr. Black straight line is exponential fit.

microcolony.

C. Microscopy

Imaging was perfomed with a Nikon Eclipse TE2000 inverted microscope equipped with a 37°C incubation chamber, automated stage (Marszhauser) and CCD camera (Coolsnap, Roper Scientific). Phase contrast images were taken automatically by imaging software Metamorph (RoperScientific) with 100X magnification objective lens every 4 minutes. An additional magnification of 1.5X was used. Light from a xenon lamp (Lambda LS) is filtered by a HCRed filter (Chroma). Care was taken to obtain the same growth rate with and without fluorescence imaging, by limiting the fluorescence image frequency to once every 12 min. Depending on the growth medium and the experimental purpose, typical experiments lasted from 5 to 16 hours. Elementary

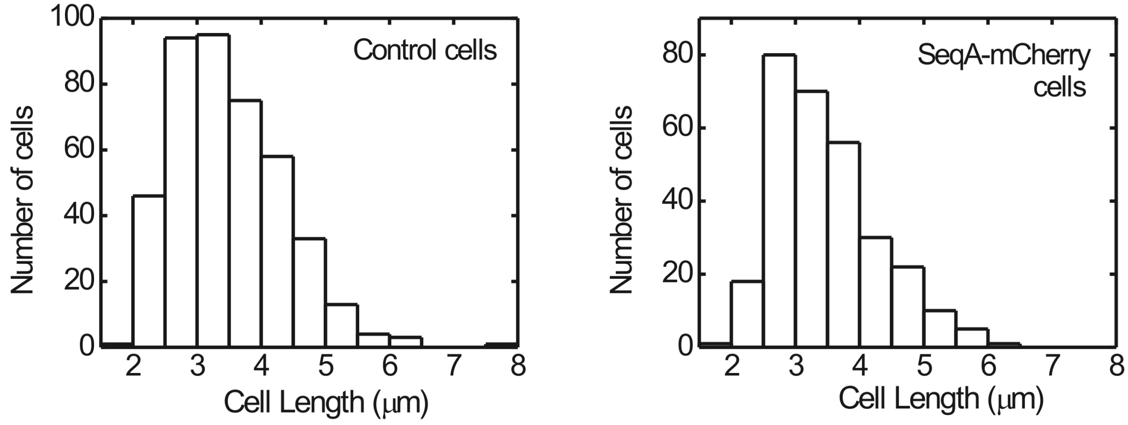

Supplementary Figure S2. **Histogram of cell lengths.** Length data of control cells expressing no SeqA-mCherry (ASC129, left panel) and cells expressing low amount of SeqA-mCherry protein (ASC215, right panel) grown exponentially in MOPS succinate medium. The data show similar cell length distibution. A Kolmogorov-Smirnov test indicated that there was no significant difference between the two data sets (D=0.068, P=0.39).

image manipulation and viewing was done with Metamorph, ImageJ and IrfanView. Cell fluorescence quantification and lineage tree construction was performed with Schnitzcell, a program written in MATLAB [4].

S2. MINIMAL MODELS OF DIVISION CONTROL

This section gives a minimal description of the problem of size homeostasis in terms of an autoregressive model, defining a framework to review the standard arguments about of size-based cell division control [5], and defines the problem of size homeostasis for multiple cell cycle sub-periods.

A. Background

Consider the simple auto-regressive discrete-time stochastic process defined by the iterative equation [6]

$$y(i) = a_0 + a_1 y(i-1) + \varepsilon \ , \tag{S1}$$

where i is a discrete variable counting time, $y(i)$ is a real variable, and ε is a zero-mean random variable, with variance σ_ε. We will be interested in the case where $y > 0$ and $a_1 > 0$. One can imagine that the noise is also Gaussian. Note that the same process can be written as a discrete Langevin equation

$$y(i) - y(i-1) = a_0 + (a_1 - 1)y(i-1) + \varepsilon \ ,$$

which looks more familiar to a physicist. It is well known that this equation leads to a steady state for y if $a_1 < 1$. For $a_1 = 1$ this is a random walk with drift a_0, and for $a_1 > 1$ there is a repulsive potential (hence the distribution of y is not expected to reach a steady state in neither cases).

The moments of the distribution of y can be calculated using properties of the geometric series. For example

$$\langle y(i) | y(i-1) \rangle = a_0 + a_1 y(i-1) \ ,$$

which, by iteration, gives for large i

$$< y > \simeq a_0 \sum_{i=0}^{\infty} (a_1^i) = \frac{a_0}{1 - a_1}$$

Analogously,

$$\sigma_y^2 = \sigma_\varepsilon^2 (1 + a_1^2 + a_1^4 + ...) = \frac{\sigma_\varepsilon^2}{1 - a_1^2} \ . \tag{S2}$$

In both the above cases, the series converge if $a_1 < 1$. In these cases, it can be seen that also higher moments converge, and the fluctuations of y are controlled. For $a_1 \geq 1$, the first two moments do not converge, hence the probability ditribution for y cannot reach a steady state. The following will use this model to produce simple arguments in the context of cell size homeostasis.

B. Timer arguments for cell-size homeostasis.

This section reviews standard arguments on size homeostasis for a timer-controlled division cycle, using the framework just described.

Exponential single-cell growth Considering the case of exponential growth within the cell cycle, and assuming that the elongation rate μ is a constant, then the cell size follows the law $L(t) = L_0 2^{\mu t} = L_0 e^{\lambda t}$ describing size L at age t. For ease of notation we will use λ instead of μ (used in the main text) in the following. For the i-th cell cycle, the size at the division time τ is

$$L_f(i) = L(i, \tau) = L_0(i) e^{\lambda \tau} \ .$$

This leads to consider the dynamics of the initial size across cell cycles, labelled by i as an auto-regressive process

$$L_0(i+1) = \frac{L_f(i)}{2} = L_0(u) e^{\lambda \tau}$$

and assuming that

$$\tau = \langle \tau \rangle + \xi \ ,$$

where ξ is a (zero mean, Gaussian) noise, one obtains the following equation for $q(i) = \log(L_0(i))$

$$q(i+1) = q(i) - \log 2 + \lambda \langle \tau \rangle + \lambda \xi \ . \tag{S3}$$

This model is a particular case of timer, since the division event is independent of any other cell cycle event. The properties of Eq. (S1) lead to conclude that a steady state can never be reached, i.e. a timer does not guarantee cell size homeostasis. If $\lambda \langle \tau \rangle = \log 2$ initial cell size makes a multiplicative random walk. If $\lambda \langle \tau \rangle$ is lower or higher than this threshold one has indefinite shrinkage, or growth of initial size, respectively. In all these cases we can say that there is no homeostasis of cell size [5], unless this is implemented by additional (extrinsic) constraints on size [7].

Linear single-cell growth. A similar argument shows that in case of single-cell elongation that is *linear* in time, a timer can guarantee size homeostasis. Indeed, if

$$L_f(i) = L_0 + \lambda \tau \ ,$$

and, as above, one assumes

$$\tau = \langle\tau\rangle + \xi ,$$

then the discrete-time process looks like

$$L_0(i+1) = \frac{1}{2}\left(L_0 + \lambda\langle\tau\rangle + \lambda\xi\right) , \tag{S4}$$

which always converges, since $a_1 = 1/2$. Note that with this notation $\langle L_0\rangle = \lambda\langle\tau\rangle$, so this formalism does not impose a constraint for the mean division rate.

Simple considerations on size homeostasis for cell-cycle sub-periods

We now set the framework for the case where the cell cycle is divided into sub-periods. We are interested in the case of three sub-periods which we call B, C, and D, and we suppose that binary cell division occurs at the end of D. If B, C and D are all regulated by timers, then we can suppose that T_B and T_C and T_D are Gaussian random variables. Then $\tau = T_B + T_C + T_D = \langle\tau\rangle + \xi$ is also a Gaussian random variable and the above argument for the timer applies. One can then conclude that there is no size homeostasis.

Suppose now that there are only two sub-periods, B and Z (which includes C and D), and Z is a regulated by a timer, but B is set by an absolute *sizer*, i.e. this sub-period ends when a target size $L_B e^{\nu}$ is reached (where ν is a Gaussian random variable of unit average defining an uncertainty on the target size). Then

$$L_0(i+1) = \frac{1}{2}\left(L_B e^{\nu}\right) e^{\lambda(T_Z+\xi_Z)} ,$$

which for the $q = \log(L_0)$ gives,

$$q(i+1) = q_B - \log 2 + \lambda T_Z + \nu + \lambda\xi_Z ,$$

in other words initial size has lognormal fluctuations around a size that is "pinned" by L_B (precisely, it is $\frac{L_B}{2}e^{\lambda T_Q}$), and the amplitude of the fluctuations is summed between uncertainty of the

sizer and fluctuations of the Q period. Additionally, one has that

$$L_B e^{\nu} = L_0 e^{\lambda(T_B+\xi_B)} ,$$

i.e.,

$$\lambda \tau_B = \log(L_B) - \log(L_0) + \nu$$

which qualitatively gives the measured anti-correlation between L_0 and the duration of the B period ($\tau_B = T_B + \xi_B$). The same procedure may be applied to a three-period cell cycle, where B and D are determined by absolute sizers and C is set by a timer. Note that noise in the target size was chosen to be multiplicative in order to have additive noise in the final equation. One can make the alternative choice of a target size $L_B + \nu$ and repeat the same calculations. Assuming that $L_B \gg \sigma_\nu$ one gets (expanding the logarithm) the same result, except that the contribution from ν is rescaled, ν/L_B. This makes no big difference given the available data since we have only one condition for L_B and its fluctuations are a parameter.

Importantly, an alternative scenario can be produced starting from the assumption that the mean target size for the B period is relative, i.e. it is L_B/L_0. In this case, $L_B = \beta L_0 e^{\nu}$, and size homeostasis is not achieved. This is a simple consequence of the fact that a "relative sizer" where the target final size is a linear function of the initial one

$$\frac{L_f}{L_0} = x^* ,$$

is not stable. Indeed, the above conditions implies that

$$L_0(i+1) = \frac{1}{2} L_0(i) x^* e^{\lambda \nu} ,$$

which gives,

$$q(i+1) = q(i) + \log \frac{x^*}{2} + \nu ,$$

i.e. once again a multiplicative RW with drift (and zero drift for $x^* = 2$). The same reasoning

applies when the cell cycle is divided into one extra sub-period controlled by a timer, since neither of the two mechanisms generates stability.

S3. GENERIC MODEL FOR THREE CELL CYCLE SUB-PERIODS

This section uses the same arguments in a more generic setting, considering the case of three periods B, C, D of the cell cycle based on the empirical observations.

A. General setting

The single-cell exponential growth of the i-th cycle can be decomposed into the contributions of each sub-period. The final size of the cell is then

$$L_f(i) = L_0(i)e^{\lambda T_B + \lambda T_C + \lambda T_D} = 2L_0(i+1)\ . \qquad \text{(S5)}$$

Here, $\lambda T_B = B + \xi_B$ and $\lambda T_C = C + \xi_C$ and $\lambda T_C = C + \xi_C$ are random variables (which we, by assumption, decompose in a deterministic part B, C, D plus Gaussian zero-mean fluctuations ξ_j, j =B,C,D). These variables are uncorrelated with the size at the start of the period if B, C, D are constants. Making B and D functions of size at the start of the period introduces a correlation. Note that B, C, D represent the mean elongation in the respective sub-periods.

As discussed in the main text, there is substantial variability in the elongation λT_j in every period j =B,C,D. Additionally, the elongation in the B and D period correlate with the cell size at the period entry (Figure 4B). We realize this correlation by making B and C functions of (logarithmic) size at entry. On the other hand, the duration of the C period is essentially independent from cell size at the entry of this period (Figure 2 and Figure 4B, center plot), hence we assume that C is a constant. By Taylor expansion, the dependency of λT_B on the logarithm of initial size L_0, and of λT_D with logarithm of the termination size L_f can be approximated as linear relations

(Figure 4B). This translates into the following equations for the net growth

$$\lambda T_B = B(\log(L_0)) + \xi_B = B_0 - k1(\log(L_0) - \langle \log(L_0) \rangle) + \xi_B$$
$$\lambda T_C = C_0 + \xi_C$$
$$\lambda T_D = D(\log(L_t)) + \xi_D = D_0 - k2(\log(L_t) - \langle \log(L_t) \rangle) + \xi_D, \tag{S6}$$

The positive constant k_1 can be estimated from data. It represents the strength of control at initiation, and can be simply estimated as the slope of the linear fit in the scatter plot of λT_B vs $\log(L_0)$ (Figure 4B, left plot). Analogously, the strength of control at division k_2 is estimated from the slope of the linear fit in the scatter plot of λT_B vs $\log(L_0)$ (Figure 4B, right plot). The Gaussian noise sets the level of fluctuations, and thus the scatter around the deterministic behaviour. This noise contribution to the coefficient of variation in the elongation is generally attributed to molecular noise [8]. To set the range of the possible values of the two parameters k_1 and k_2, we can consider the limit cases of no control (or "timer") and of an absolute sizer. In the case of a timer, the size-growth plots would show no correlation (and thus necessarly $k_1 = k_2 = 0$). On the other hand, an absolute sizer, i.e. a fixed size threshold that have to be reached to proceed in the next cell cycle period, would imply a -1 slope of the linear fit.

Once the values of k_1 and k_2 are fixed, Equations S5 and S6 define an auto-regressive map, from which we set out to evaluate fluctuations in the initial size L_0. Once again, it is more convenient to work with the variable $q = \log L_0$. We ask whether a steady state with fixed-point mean $\langle q \rangle = q^*$ can exist, and we therefore assume this condition and verify its consistency. We can interpret Equation (S6) as a Taylor expansion around the hypothetical fixed point q^*.

The net elongation in the D period can be expressed as a function of the initial size, noticing that $\log(L_f) = \log(L_0) + B(\log(L_0)) + C_0 + \xi_{BC}$, with $\xi_{BC} = \xi_B + \xi_C$. Thus,

$$\lambda T_D = D_0 - k_2(1 - k_1)(q - q^*) - k_2 \xi_{BC} + \xi_D. \tag{S7}$$

Starting from Equation S5, the stochastic process defining the initial size distribution gives the

auto-regressive AR(1) process

$$
\begin{aligned}
q(i+1) &= q(i) - \log(2) + B(q - q^*) + C_0 + D(q) + \xi_{BCD} \\
&= q(i) - \log(2) + B_0 + D_0 + C_0 - [k_1 + k_2(1-k_1)](q - q*) + (1-k_2)\xi_B + (1-k_2)\xi_C + \xi_D \\
&= q(i) - [k_1 + k_2(1-k_1)](q - q*) + (1-k_2)\xi_B + (1-k_2)\xi_C + \xi_D \, . \qquad \text{(S8)}
\end{aligned}
$$

Where $\xi_{BCD} = \xi_B + \xi_C + \xi_D$, and we have used the identity $B_0 + D_0 + C_0 = \langle \lambda(T_B + T_C + T_D) \rangle = \log(2)$, which is a necessary condition for a stationary size distribution. One can also verify that the parameters B_0, C_0 and D_0 correspond to the average elongations in the respective periods. Note that having used the logarithm of size to define the AR(1) process naturally implies a lognormal stationary distribution of sizes, which is compatible with empirical data (Figure 4C).

The variance of q at steady state can be calculated using Equation (S2), as

$$
\sigma_q^2 = \frac{(1-k_2)^2 \sigma_{\xi_B}^2 + (1-k_2)^2 \sigma_{\xi_C}^2 + \sigma_{\xi_D}^2}{(k_1 + k_2(1-k_1))(2 - k_1 - k_2(1-k_1))} \, . \qquad \text{(S9)}
$$

The above expression shows how size fluctuations result from the interplay between intrinsic fluctuations in the B, C, and D period and the control mechanism. The general condition for stationarity is given by the expression

$$
-\frac{k_1}{1-k_1} < k_2 < \frac{2-k_1}{1-k_1} \qquad \text{(S10)}
$$

which, given the physical range of parameter values for k_1 and k_2 in $[0, 1]$, simply states that at least in one of the two periods there must be a size control, i.e., a negative correlation between size and elongation, in order to achieve size homeostasis. Equation S9 describes how the fluctuations in growth in the three periods propagate in order to build the variance of the stationary size distribution. In particular, the role of the two controls defined by k_1 and k_2 is shown in Figure 4A. The minimum variance of Equation S9 is achieved for $k_2 = 1$, meaning that, within this framework, the best error correction is achieved by an absolute sizer at division, regardless of the level of control at initiation. These results also show that (to linear order) the existence of an uncontrolled C period cannot affect convergence, i.e., any nonzero correlation theoretically triggers cell homeostasis in size (although the steady state may well be unphysical if the spread in logarithmic size is too high,

e.g., compared to the mean).

Finally, the variance of initial size can be expressed as a function of the variance in the elongation in the three periods, which are measured in our data with respect to the gaussian noises ξ_B, ξ_C, ξ_D. The expression of the three variances can be calculated straightforwardly as

$$\begin{aligned}\sigma_B^2 &= \langle(\lambda T_B)^2\rangle - \langle\lambda T_B\rangle^2 = \sigma_{\xi_B}^2 + k_1^2\sigma_q^2 \\ \sigma_C^2 &= \sigma_{\xi_C}^2 \\ \sigma_D^2 &= \langle(\lambda T_D)^2\rangle - \langle\lambda T_D\rangle^2 = \sigma_{\xi_D}^2 + k_2^2(\sigma_B^2 + \sigma_C^2) + k_2^2(1-2k_1)\sigma_q^2 \,,\end{aligned} \tag{S11}$$

and then substitute in Equation S9

$$\sigma_q^2 = \frac{1}{2}\frac{(1-2k_2)(\sigma_B^2+\sigma_C^2)+\sigma_D^2}{k_1+k_2(1-2k_1)} \,. \tag{S12}$$

B. Compatibility between model and data

The modeling framework described above generates some predictions about the relation between observable variables. These can be tested using the empirical data. In particular, a standard test of the presence of size control is the presence of a negative correlation in the size-growth plot of $\lambda\tau$ vs $\log(L_0)$ (Figure 4D). Given the estimated values of the control strengths k_1 and k_2 in the two sub-periods, the model constrains the expected slope of the linear fit of this size-growth plot. More specifically, the predictions given by the following equation

$$\lambda\tau = \lambda(T_B+T_C+T_D) = B_0 - k_1(q-q^*) + C_0 + D_0 - k_2(1-k1)(q-q^*) - k_2\xi_{BC} + \xi_{BCD}. \tag{S13}$$

Therefore, the linear fit is expected to have slope $-[k_1 + k_2(1-k_1)]$, and similarly the intercept value can be estimated. Figure 4D compares the scatter plot with the predicted linear fit (dashed red line), showing a good compatibility with the empirical data. Additionally, one can verify the compatibility between the birth size distribution and the model prediction (Equation S12). Figure 4C shows the good agreement between model (continuous red line) and experimental data

(histogram).

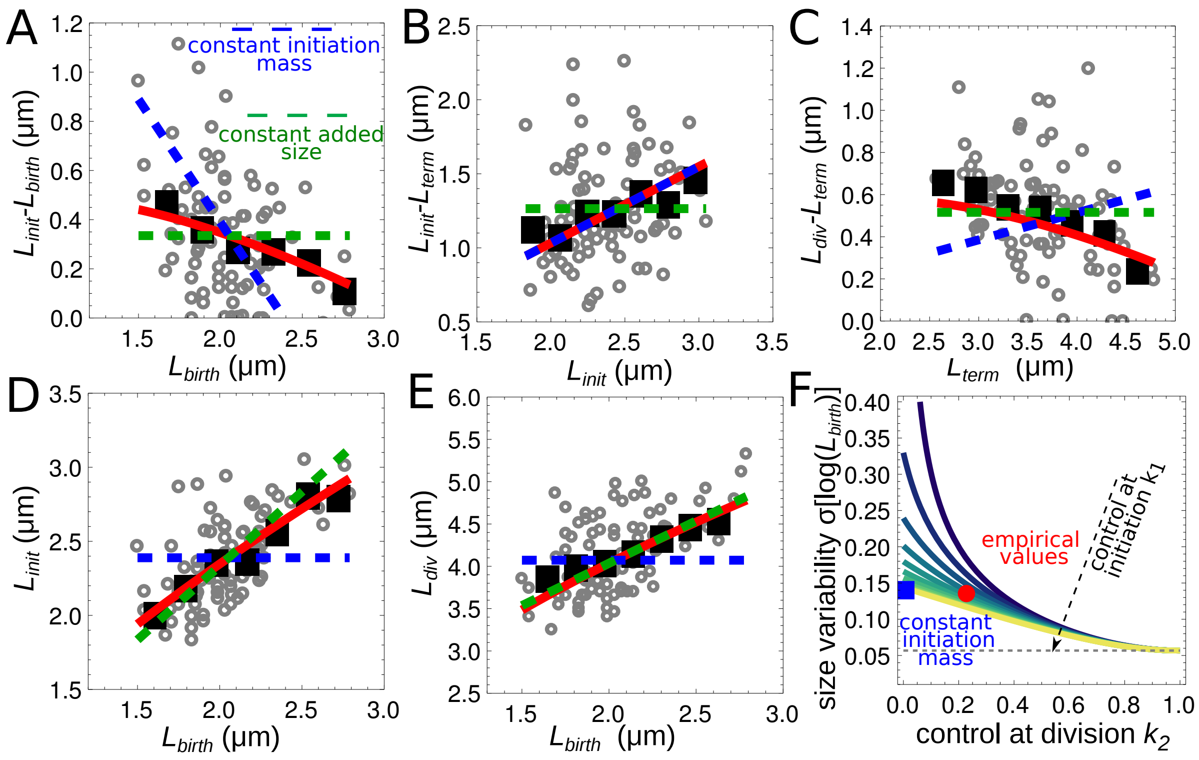

Supplementary Figure S3. **Comparison with alternative models.** A, B, and C) Added length in each sub-period against the length at the beginning of the sub-period. Black squares are binned averages. Green dashed line: model assuming a constant added size in each period. Blue dashed line: constant initiation mass model. D) Cell length at initiation against cell length at birth. E) Cell length at division against cell length at birth. F) Predicted birth size variability given strength of compensation in the B period (k_1) and in the D period (k_2), and the parameter values for the constant initiation size model (blue square), and the fit to our data (red point). see Section S3 for details.

---

[1] A. M. Adiciptaningrum, I. C. Blomfield, and S. J. Tans, "Direct observation of type 1 fimbrial switching.," *EMBO Rep*, vol. 10, pp. 527–532, May 2009.

[2] T. Brendler, J. Sawitzke, K. Sergueev, and S. Austin, "A case for sliding SeqA tracts at anchored replication forks during Escherichia coli chromosome replication and segregation.," *EMBO J*, vol. 19,

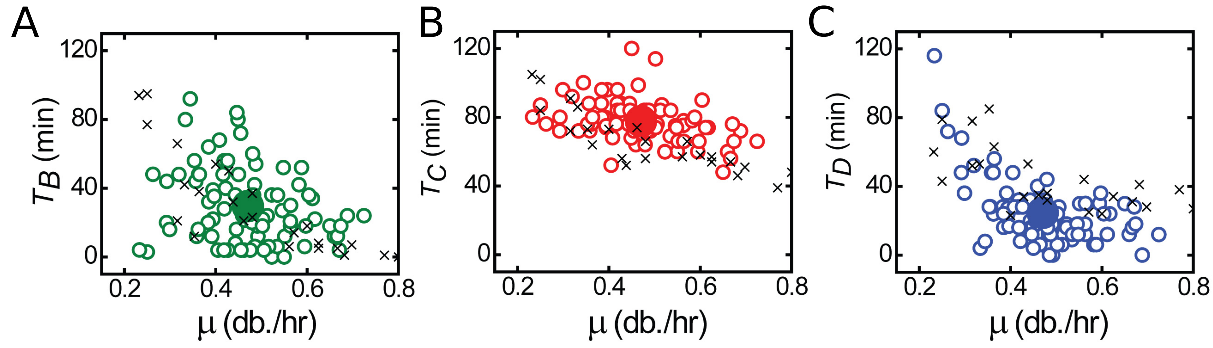

Supplementary Figure S4. **Comparison with bulk measurements of B,C and D period duration.** Comparison with bulk measurements of B,C and D period duration, respectively in panels A, B, and C. Open circles: our single-cell data. Filled circles: mean values of our single-cell data. Crosses: population-mean values obtained for different growth conditions and genetic backgrounds (see ref. 3).

pp. 6249–6258, Nov 2000.

[3] N. C. Shaner, P. A. Steinbach, and R. Y. Tsien, "A guide to choosing fluorescent proteins.," *Nat Methods*, vol. 2, pp. 905–909, Dec 2005.

[4] N. Rosenfeld, J. W. Young, U. Alon, P. S. Swain, and M. B. Elowitz, "Gene regulation at the single-cell level.," *Science*, vol. 307, pp. 1962–1965, Mar 2005.

[5] J. M. Skotheim, "Cell growth and cell cycle control.," *Mol Biol Cell*, vol. 24, p. 678, Mar 2013.

[6] T. C. Mills, *Time series techniques for economists*. Cambridge: Cambridge University Press, 1990.

[7] L. Robert, M. Hoffmann, N. Krell, S. Aymerich, J. Robert, and M. Doumic, "Division in escherichia coli is triggered by a size-sensing rather than a timing mechanism.," *BMC Biol*, vol. 12, p. 17, 2014.

[8] S. D. Talia, J. M. Skotheim, J. M. Bean, E. D. Siggia, and F. R. Cross, "The effects of molecular noise and size control on variability in the budding yeast cell cycle.," *Nature*, vol. 448, pp. 947–951, Aug 2007.
